# On-demand electrically controlled melatonin release from PEDOT/SNP composite improves quality of chronic neural recording

**DOI:** 10.3389/fbioe.2023.1284927

**Published:** 2023-11-15

**Authors:** Ying Zhu, Yuxi Yang, Gongang Ni, Shijin Li, Wei Liu, Zhongbao Gao, Xiao Zhang, Qi Zhang, Changyong Wang, Jin Zhou

**Affiliations:** Beijing Institute of Basic Medical Sciences, Beijing, China

**Keywords:** implantable brain-computer interface, PEDOT/SNP-MT coating, electrochemical impedance, CNT electrodes, neural recording

## Abstract

Long-time and high-quality signal acquisition performance from implantable electrodes is the key to establish stable and efficient brain-computer interface (BCI) connections. The chronic performance of implantable electrodes is hindered by the inflammatory response of brain tissue. In order to solve the material limitation of biological interface electrodes, we designed sulfonated silica nanoparticles (SNPs) as the dopant of Poly (3,4-ethylenedioxythiophene) (PEDOT) to modify the implantable electrodes. In this work, melatonin (MT) loaded SNPs were incorporated in PEDOT via electrochemical deposition on nickel-chromium (Ni-Cr) alloy electrode and carbon nanotube (CNT) fiber electrodes, without affecting the acute neural signal recording capacity. After coating with PEDOT/SNP-MT, the charge storage capacity of both electrodes was significantly increased, and the electrochemical impedance at 1 kHz of the Ni-Cr alloy electrodes was significantly reduced, while that of the CNT electrodes was significantly increased. In addition, this study inspected the effect of electrically triggered MT release every other day on the quality and longevity of neural recording from implanted neural electrodes in rat hippocampus for 1 month. Both MT modified Ni-Cr alloy electrodes and CNT electrodes showed significantly higher spike amplitude after 26-day recording. Significantly, the histological studies showed that the number of astrocytes around the implanted Ni-Cr alloy electrodes was significantly reduced after MT release. These results demonstrate the potent outcome of PEDOT/SNP-MT treatment in improving the chronic neural recording quality possibly through its anti-inflammatory property.

## 1 Introduction

The establishment of a stable and efficient brain-computer interface (BCI) connection ensures the reliability of the neural interface device, which influences by the electrodes in contact with the brain tissue. BCI instruments using implantable microelectrodes have become an important tool in neuroscience investigation because of their higher spatial and temporal resolution (Zhang and Lieber, 2016; Wang et al., 2018; Won et al., 2018). These microelectrodes can communicate between BCI instruments and the neural tissue by detecting or stimulating neural activity, demonstrating the great potential of BCI in clinical application (Irwin et al., 2017; Pei and Tian, 2020; Kwon et al., 2021).

Obtaining excellent long-term signal from implantable microelectrodes is one of the most important issues in neuroscience research. The amplitude of the single unit recording decreased significantly over time, which restrict their chronic and clinical applications (Keefer et al., 2008; Kozai et al., 2016; Golabchi et al., 2018). There are at least two major reasons that may limit their long-term use (Hejazi et al., 2021). Firstly, the implantation of electrodes will evoke inflammatory response of tissues in the host body (Zhou et al., 2013; Hejazi et al., 2021). Tissue inflammation leads to neuronal death and the formation of glial scars, resulting in changes in the properties of the electrodes (Rivnay et al., 2017; Chen et al., 2021; Hejazi et al., 2021). Secondly, the recording quality of the microelectrodes is affected by physical instability. Corrosion and degradation of the microelectrode material have been observed for a long time, which limit their long-term use and clinical application (Liu et al., 2011; Hejazi et al., 2021). Carbon-based microfiber electrodes, such as CNT electrodes, with better compliance with surrounding tissues, smaller tissue inflammation responses, have been selected as the microelectrode materials for BCI research with better chemical and mechanical stability compared to other electrodes (Green et al., 2008; Zhang et al., 2018; Lu et al., 2019). Although microfiber electrodes are more suitable for chronic neural recording, additional surface modifications are often required to improve their ability to be used for neural recording or stimulation (Zhou et al., 2013; Kim et al., 2017; Jayaram et al., 2019; Saunier et al., 2020).

MT is an attractive candidate among the multiple treatments for tissue inflammatory response and neuronal death (Mayo et al., 1998; Sainz et al., 2003; Banach et al., 2011). As an antioxidant, MT has a significant effect on inhibiting neuroinflammation and the resulting neuronal death (Carrillo-Vico et al., 2013; Mauriz et al., 2013; Hardeland, 2021). Meanwhile, MT can play a neuroprotective role in the treatment of neurological diseases (Korkmaz et al., 2009; Permpoonputtana and Govitrapong, 2013; Liu et al., 2019; Gao et al., 2020; Hu et al., 2021). In terms of neural interface applications, recent research has shown that MT can protect the health of neurons around the implanted nerve electrodes and maintain chronic recording quality when it is administered systemically every day (Golabchi et al., 2018). However, due to the large amount of drug injected intraperitoneally and low efficiency in brain entry, long-term use of MT may lead to many side effects. Studies have shown that if exogenous MT is used under the condition of normal secretion of MT or a large amount of MT is eaten for a long time, there may be certain side effects, such as morning drowsiness, vivid dreams, headache and dizziness (Korkmaz et al., 2009; Jung-Hynes et al., 2010; Rossignol and Frye, 2011; Porfirio et al., 2017; Posadzki et al., 2018; Xu et al., 2020; Duan et al., 2021). Therefore, continuous systemic MT delivery is not a desirable choice and a locally controlled administration strategy should be established.

Conductive polymer coatings provide an ideal alternative. The recording performance of nerve microelectrodes can be improved by increasing the effective surface area and decreasing the impedance with conductive polymers (Poole-Warren et al., 2010; Liu et al., 2011; Ludwig et al., 2011; Saunier et al., 2020; Yang et al., 2021). Conductive polymers can be electropolymerized on microelectrodes with positively charged polymer backbone, attracting negatively charged dopants in the film. When a sufficient negative current is applied, the dopant molecules may be released. Because of this unique character, conductive polymers are used to deliver drug by electrically controlled release (Abidian et al., 2006; Thompson et al., 2011; Wang et al., 2018). These findings suggest the possibility of local delivery of MT from nerve electrodes. However, MT delivery by conductive polymer is difficult to complete because of the electrochemical instability property (Wang et al., 2004). Recently, silicon-based sulfonated nanoparticles have been developed to deliver drug. SNPs not only increase the amount of the loading drug, but also can protect electroactive drugs such as MT from losing its redox efficacy during the electropolymerization process (Woeppel et al., 2019; Tan et al., 2021).

**FIGURE 1:**
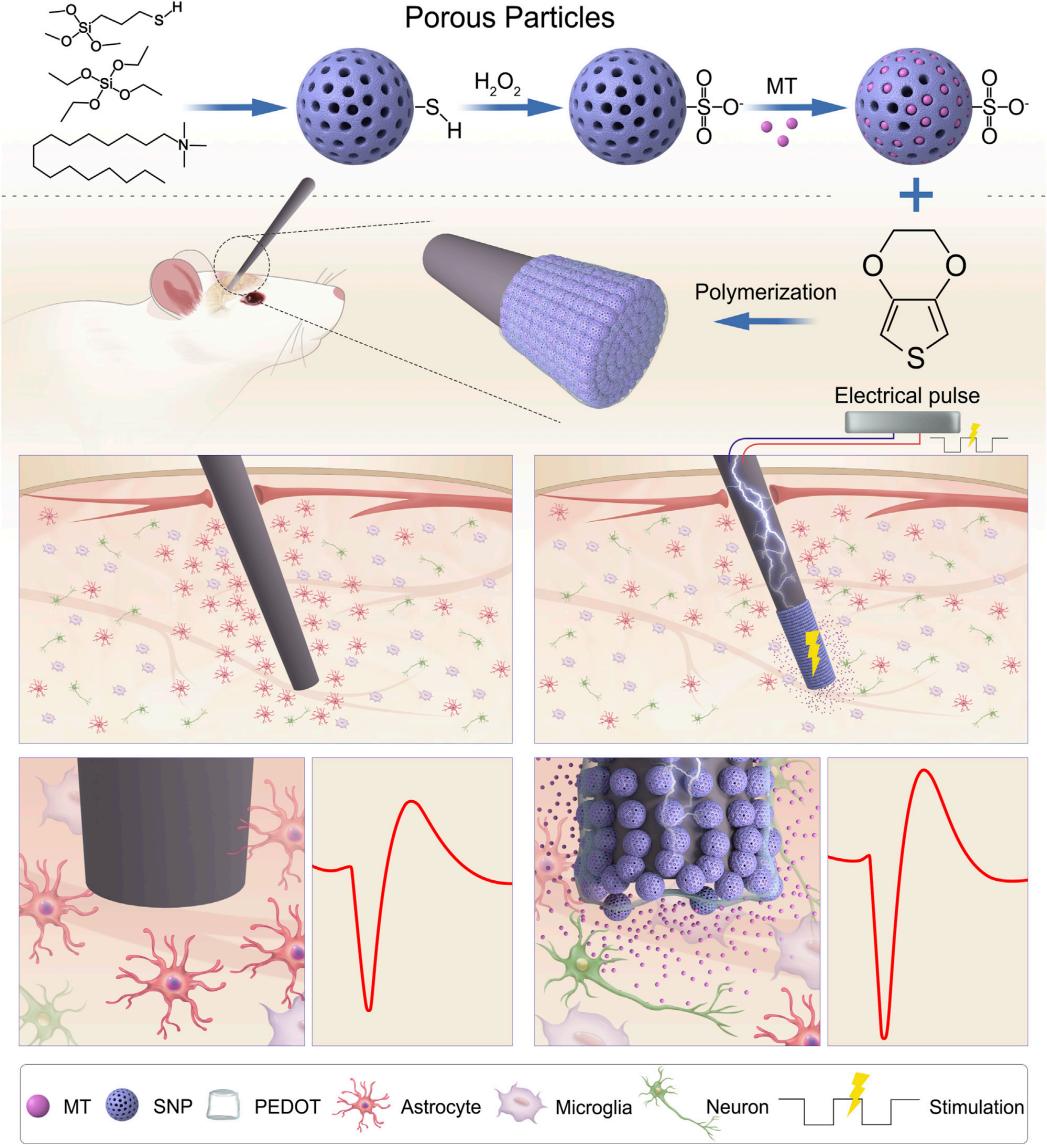
Schematic illustration of PEDOT/SNP(MT) coated electrode for improving quality of chronic neural recording. Melatonin Loading porous SNPs were used as dopants for PEDOT films by electropolymerization. After loading with MT in the electrode membrane, MT is delivered directly to the implantation site as needed by a unidirectional rectangular waveform which can reduce the number of astrocytes around the implanted electrodes and improve the chronic neural recording quality. MT: melatonin; SNP: sulfonated silica nanoparticle.

In this work, the loading and releasing of MT from PEDOT/SNP coated microelectrodes were investigated (Figure 1). The electrochemical properties of the electrodes, including electrochemical impedance and charge storage capacity were analyzed. After PEDOT/SNP-MT coating, the acute neural signal recording capacity of Ni-Cr alloy electrode and CNT fiber electrodes was not affected. In addition, both MT modified Ni-Cr alloy electrodes and CNT electrodes showed significantly higher spike amplitude after 26-day recording, and the histological studies showed that the number of astrocytes around the implanted Ni-Cr alloy electrodes was significantly reduced after MT release. These results indicate that PEDOT/SNP-MT film can improve the stability of the electrodes and has a good application prospect.

## 2 Materials and methods

### 2.1 Materials and properties

CNT fiber electrodes were provided by the research group of Duan Xiaojie, Institute of Technology, Peking University. A-M Systems (United States) obtained Ni-Gr alloy electrode with a diameter of 35 μm. CNT fibers were spun from spinnable CNT array, which was grown by chemical vapor deposition as described previously (Lu et al., 2019). Scissors were used to cut the insulated CNT fiber into the required length.

### 2.2 Preparation of drug-loaded nanoparticles

The synthesis of porous SNP was based on previous reports (Woeppel et al., 2019; Tan et al., 2021). Briefly, a solution comprising of deionized water (36 mL), ethanol (5 mL), CTAB (1.3 g) and triethanolamine (6.2 mL) were mixed, heated to 60°C, and stirred for 30 min. After that, 3 mL TEOS and 1 mL MTS were added dropwise, 100 µL MTS was added 1 h later, and cooled after 2 h of reaction. The particles were collected by centrifuge (20000 rpm × 10 min). The particles were then washed with water and ethanol one after the other to remove the surfactant template, and the ethanol (100 mL) containing the particles and HCl (2.5 M) were mixed and heated under reflux at 70°C overnight; The particles were collected and washed with water. Two grams of collected particles were suspended in H_2_O_2_ (20%, 25 mL) and H_2_SO_4_ (20 µL) was then added. After oxidation, the particles were collected by centrifugation and washed with water and stored until use at 4°C.

The MT compound (25 mg mL^–1^) was dissolved in ethanol; Then 5 mg SNP was weighed, mixed with 200 μL MT solution, and sonicated for 20 min to load drug. Drug Loading particles were collected by centrifuge and dried under vacuum. SEM images were taken by JSM-7001F.

### 2.3 Electropolymerization and MT release

Electrodeposition experiments were performed using a CHI660D electrochemical workstation (CH Instruments, United States). A total of 20 mg SNP was suspended in 20 mL EDOT (0.01 M) aqueous solution. Electropolymerization was carried out for 200 s on Ni-Gr alloy electrode or CNT fiber electrode, the polymerization current intensity was set to 10 nA. After electropolymerization, the electrodes were then washed with PBS for 2 h and then soaked overnight to remove any adsorbed drugs. Drug release was performed with a unidirectional rectangular waveform (5 ms, −10 µA, 100 Hz) into 250 μL of PBS or the animal model using PlexStim Electrical Stimulator. The concentration of the MT solution is measured by UV-visible absorption.

### 2.4 Electrochemical characterization

Electrochemical impedance spectroscopy (EIS) and cyclic voltammetry (CV) measurements were performed using a CHI660D electrochemical workstation with a three-electrode configuration. EIS was carried out in the range of 1–100 kHz. CV were scanned between −0.6 V and 0.8 V at a rate of 50 mV/s. The cathodic charge storage capacity (CSCc) was determined as the time integral of the recorded negative current. The electrodes after the deposition of the PEDOT film were immersed in PBS for 30 min and cyclically scanned multiple times before each CV was recorded to ensure that the deposited PEDOT film reached a stable state. Results from all electrochemical data are the average of 8 samples under each experimental condition.

### 2.5 Animal surgery and electrodes implantation

Adult Sprague-Dawley rats (250–320 g, Experimental Animal Center, Academy of Military Medical Science) were used throughout this study. All animal procedures complied with the guidelines of the Recommendations from the Declaration of Helsinki and were approved by the Institutional Animal Care and Use Committee of the Chinese Academy of Military Medical Science. Rats were anesthetized with 5% isoflurane and secured in a stereotactic apparatus (RWD Life Science, United States). The hair on the rats’ head was shaved, then the exposed skin was disinfected with iodine. The scalp was then cut open to expose the clean skull, and three sterilized stainless steel screws were screwed into the skull to serve as grounding screw and bone anchor. A craniotomy (2–3 mm in diameter) was performed directly above the area of interest. Dura mater was removed at the craniotomy sites for electrode implantation.

Tungsten wire with a diameter of 50 μm was used to help implant CNT fiber electrodes. Individual CNT fiber electrode was dipped in sterilized 4% polyethylene oxide (PEO) solution and dried in air to paste to the tungsten wire. The CNT fiber/tungsten complex or Ni-Cr electrode was then inserted into the craniotomy with a micromanipulator to reach the desired target area. This insertion process was controlled within 1 min to avoid the dissolution of PEO before reaching the desired depth. The tungsten wires were retracted after the insertion, leaving the CNT fiber electrodes in the brain. Upon successful implantation, craniotomies were sealed with the noncytotoxic silicone elastomer, Kwik-Sil (World Precision Instruments, United States), followed by dental acrylic.

### 2.6 *In vivo* neural recording and data analysis

1%–2.5% isoflurane was used for anesthesia during chronic neural electrical recordings in this study. Voltage signals from the neural electrodes were amplified and digitized using a plexon system (Plexon, Dallas, TX, USA) with a sampling rate of 40,000 Hz. A 250-Hz high-pass filter and a 60-Hz notch filter were applied for single-unit recordings. Spike detection and sorting were performed using Offline Sorter (Plexon, Inc.). A voltage threshold was set to be 3 times or higher of the noise level. The total waveform length to be extracted was set to be 2 ms, with prethreshold period of 0.5 ms. Valley-seeking method or manual selection was used to detect and isolate clusters of different units.

The unit amplitude was measured from peak to the trough of the mean waveform for each unit. Noise level was calculated as the standard deviation of the high-pass filtered voltage traces after the individual spike waveforms were removed. SNR was calculated as the amplitude divided by two times the noise level for each unit. The valley-to-peak time was measured as the time from the peak to the trough of the mean spike waveform.

### 2.7 Immunohistochemistry

30 days after electrode implantation, rats were anesthetized and transcardially perfused with 200 mL PBS, followed by 200 mL 4% paraformaldehyde (PFA). The brain tissue was removed and postfixed in 4% PFA for 48 h at 4°C. Then, brains were soaked in a 15% sucrose (Sigma-Aldrich Corp., United States) solution at 4°C overnight followed by a 30% sucrose solution until the tissue totally sunk to the bottom of the PBS solution. After being cryoprotected using the optimal cutting temperature (OCT) compound (Tissue-Tek, United States), the tissue sample was kept at −80°C until it was sliced. Frozen tissue was horizontally sectioned into 30 μm thick per slice using a cryostat machine (Leica CM 1950, Germany) and stored in PBS. 5 mg/mL sodium borohydride in 1 × PBS was applied to slices for 30 min and rinsed with PBS afterwards. Tissue sections were then blocked with 3% (w/v) bull serum albumin (BSA) at 4°C for 2 h and washed by PBS solution. Then slices were incubated overnight at 4°C with primary antibodies: Rabbit anti-glial fibrillary acidic protein (GFAP) (targeting astrocytes, 1:500, Abcam#ab7260, USA) and mouse anti-neuronal nuclear (NeuN) (targeting nuclei of neurons, 1:500, Millipore# MAB377, United States). After thoroughly washed, slices were incubated at room temperature for 2 h in secondary antibodies, which include: 1:200, Alexa Fluor 568 goat anti-rabbit; 1:200, Alexa Fluor 532 goat anti-mouse, Invitrogen, United States). 4′, 6-diamidino-2-phenylindole (DAPI, Sigma-Aldrich, United States) was then applied to the slices to stain cell nuclei. Then the slices were mounted onto glass slides with Prolong Gold (Invitrogen, United States) gel for later imaging. Images were taken with a Nikon Ti-E Inverted Live Cell Imaging System using ×20 magnification. Normalized fluorescence intensity of immunomarkers as a function of distance from the implant tract was calculated with Igor Pro-6.2 (WaveMetrics). The center of the implant tract was set as x = 0 μm (Supplementary Figure S6). Circular outlines of 20 μm length were segmented up to the distant uninjured regions (−200 μm away, defined as background). The average gray scale pixel intensity (0–255 a.u.) for all of the pixels in this 20 μm circular outline (bins) were calculated and normalized to the background region for each image.

### 2.8 Statistical analysis

Data are shown as mean ± SEM. The Student’s t-test was applied for statistical analysis by GraphPad Prism (version 9; GraphPad Software, La Jolla, CA) and Microsoft Excel (version 2020; Microsoft Software, Redmond, Washington) and values *p* < 0.05 was considered significant (*p < 0.05, **p < 0.01, and ***p < 0.001).

## 3 Results

### 3.1 Characterization of nanoparticles

In order to produce nanoparticles capable of doping conductive polymer films, sulfonate-modified SNPs were selected (Woeppel et al., 2019; Tan et al., 2021). Briefly, with CTAB as the surface template, porous thiol-modified nanoparticles were synthesized from tetraethyl orthosilicate (TEOS) and MTS under alkaline conditions. Thiolated nanoparticles (TNPs) were subsequently oxidized to SNP under hydrogen peroxide and sulfuric acid.

**FIGURE 2:**
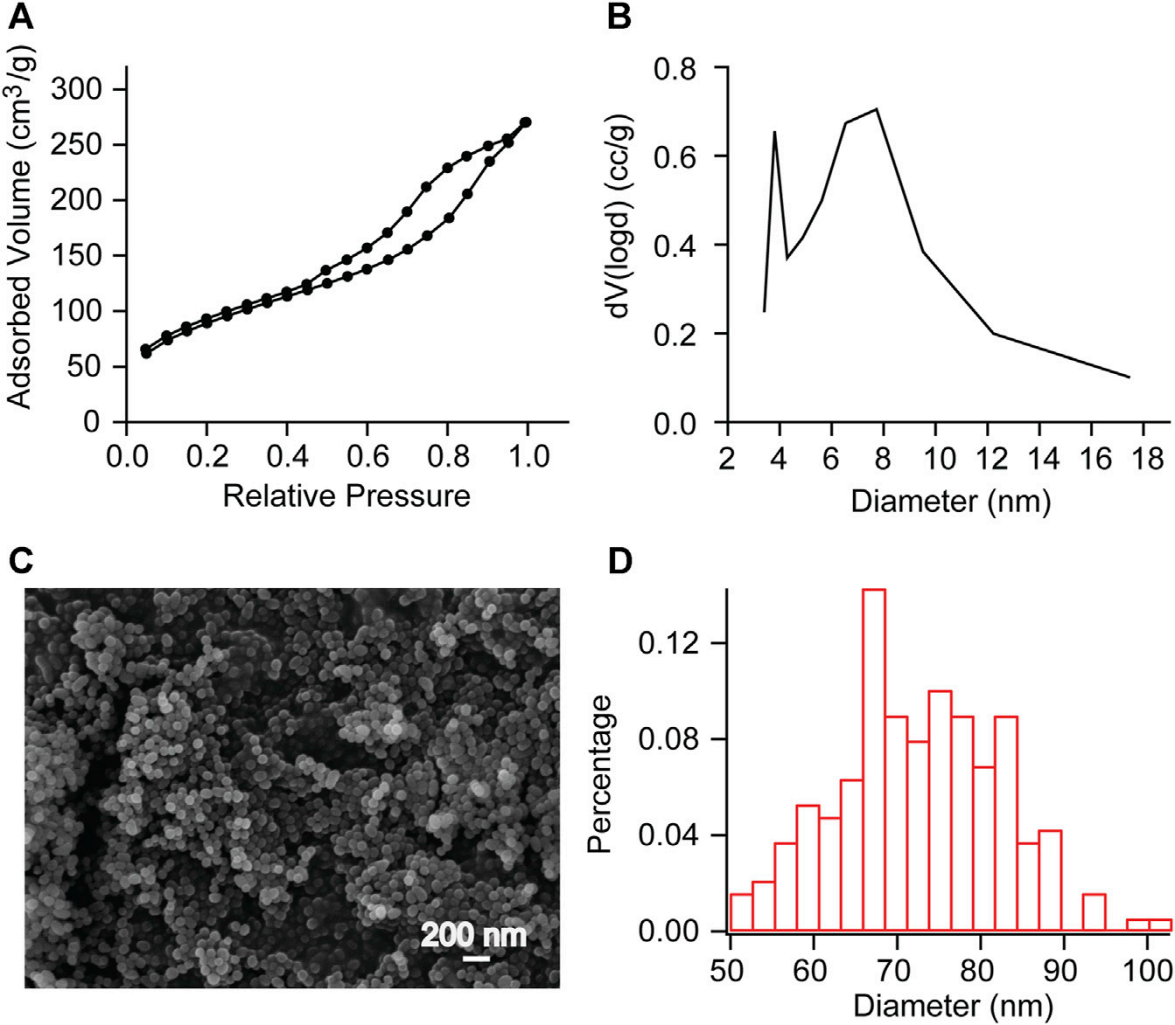
BET measurements and SEM observation of mesoporous SNP **(A, B)** Adsorption/desorption isotherms of N2 and Pore diameter calculated with DFT method. Further calculations reveal a pore volume of 0.336 cc/g and a surface area of 195.085 m2/g. **(C, D)** SEM observation showed that the average particle diameter was 72.424 nm. This indicates that we have successfully synthesized mesoporous sulfonate modified silica nanoparticles.

The pore diameter and particle diameter of the mesoporous silica nanoparticles were observed by Brunauer-Emmett-Teller (BET) method and scanning electron microscope (SEM) observation (Figure 2). The measurement results showed that the pore volume was 0.336 cc/g, the surface area was 195.085 m^2^/g, the pore diameter was between 4 and 18 nm (Figure 2B), and the average particle diameter was 72.424 nm (Figures 2C,D). This indicates that we have successfully synthesized mesoporous sulfonate modified silica nanoparticles.

### 3.2 Electropolymerization and electrochemical properties of the PEDOT/SNP-MT

SNPs loading MT were used as dopants for PEDOT films by electropolymerization. Polymerization and measurements were performed on 20 μm CNT electrodes and 35 μm Ni-Cr alloy electrodes, the sizes of which were relevant to the microelectrode design and allowed the ability of the conductive polymer to be examined as an advanced interface coating (Fairfield, 2018; Hejazi et al., 2021; Yang et al., 2021). Supplementary Figures S1, S2 showed the surface morphology of Ni-Cr alloy electrodes and CNT electrode before (Supplementary Figure S1A, Supplementary Figure S2A) and after constant current deposition (Supplementary Figure S1B, Supplementary Figure S2B). The Ni-Cr alloy electrodes surface has obvious concave-convex structure. In contrast, after modifying PEDOT/SNP-MT, it was observed that the tip of Ni-Cr alloy electrodes was covered by PEDOT films, which filled the gap of electrode tip. Compared with Ni-Cr alloy electrodes, it was observed that the CNT electrode tips were covered by aggregated nanoparticles after modification. These structures were beneficial to the contact between electrodes and nerve cells.

**FIGURE 3:**
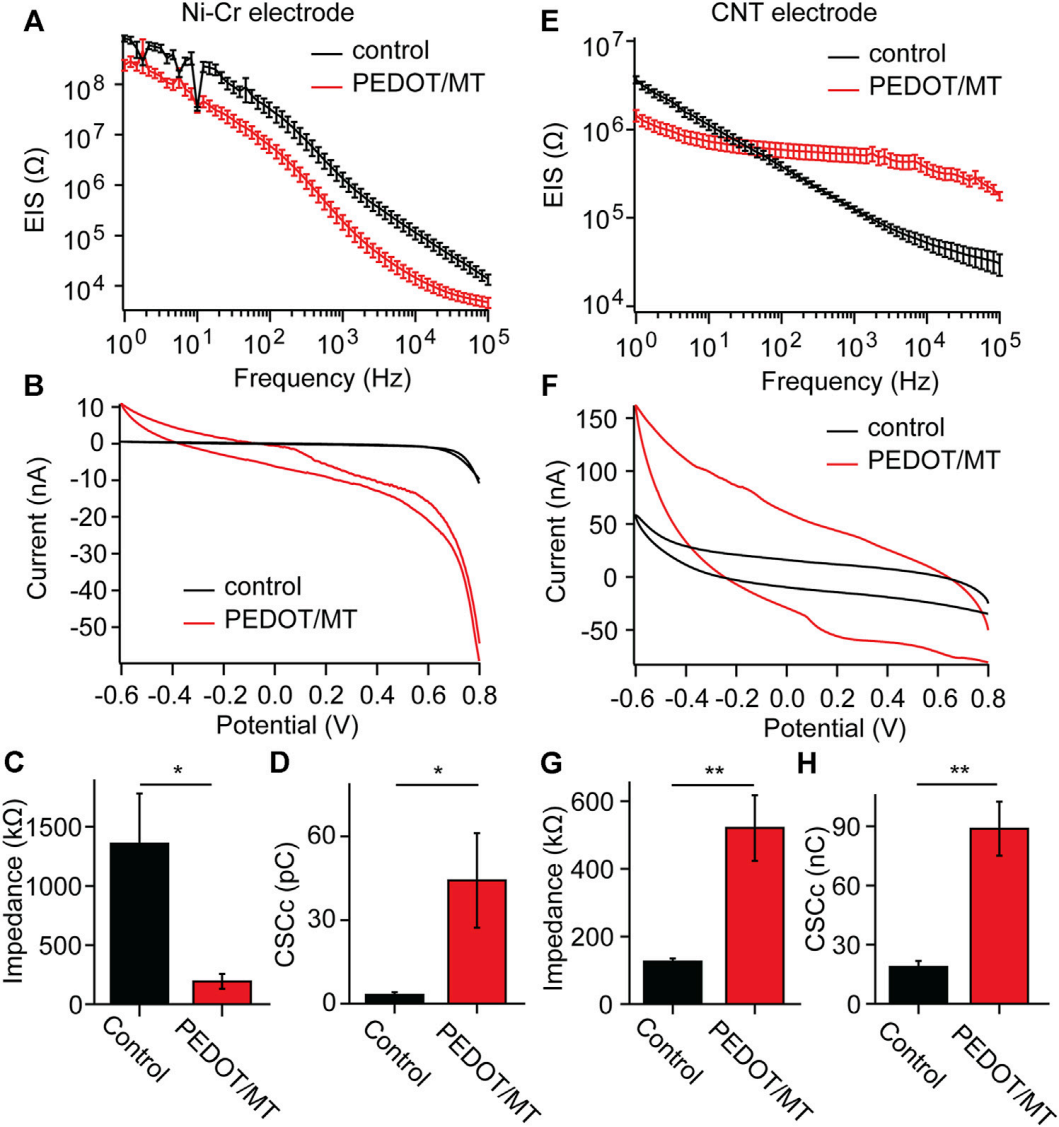
Electrochemical properties of the Ni-Cr alloy electrode and CNT fiber electrodes before and after deposition of PEDOT/SNP-MT film **(A)** Electrochemical impedance spectroscopy of Ni-Cr alloy electrodes before and after constant current deposition of PEDOT/SNP-MT film. **(B)** The voltammetric cycle curve of Ni-Cr alloy electrodes before and after constant current deposition of PEDOT/SNP-MT film. **(C)** Electrochemical impedance spectroscopy at 1 kHz before and after constant current deposition of PEDOT/SNP-MT film. **(D)** The charge storage capacity of Ni-Cr alloy electrodes before and after constant current deposition of PEDOT/SNP-MT film. **(E)** Electrochemical impedance spectroscopy of CNT fiber electrodes before and after constant current deposition of PEDOT/SNP-MT film. **(F)** The voltammetric cycle curve of CNT fiber electrodes before and after constant current deposition of PEDOT/SNP-MT film. **(G)** Electrochemical impedance spectroscopy at 1 kHz before and after constant current deposition of PEDOT/SNP-MT film. **(H)** The charge storage capacity of CNT fiber electrodes before and after constant current deposition of PEDOT/SNP-MT film. These results indicate that the electrochemical interface properties of PEDOT/SNP-MT coating electrodes are improved compared with bare electrodes. Results from all electrochemical data are the average of 8 samples under each experimental condition. Error bars show SEM. *p < 0.05, **p < 0.01, Student’s T-Test.

EIS is a powerful tool for characterizing the deposition of conductive polymers, which reflects the signal recording ability of implantable electrodes of brain-computer interface (Lu et al., 2019; Woeppel et al., 2019; Tan et al., 2021). We measured the electrochemical impedances of CNT fiber electrode and Ni-Cr alloy electrode before and after PEDOT/SNP-MT modification, and the results were shown in Figure 3. After PEDOT/SNP-MT coating, the electrochemical impedance at 1 kHz of the Ni-Cr alloy electrodes was significant reduced (From 1356.2 kΩ to 192.8 kΩ), while that of the CNT electrodes was significant increased (From 125.2 kΩ to 520.5 kΩ) (Figures 3A, C, E, G), this may be due to the higher electrochemical impedance of Ni-Cr alloy electrode and the better conductivity of CNT electrode relative to conductive polymer (Zhou et al., 2013; Saunier et al., 2020; Hejazi et al., 2021). The voltammetric cycle curve has been widely used to evaluate the redox performance and charge storage capacity (CSC) of nerve electrodes (Kros et al., 2005; Liu et al., 2011; Zeng et al., 2022). In this study, the scanning rate was 50 mV s^−1^, and the scanning potential was limited within the range of −0.6–0.8 V. After PEDOT/SNP-MT modification, the surrounded area of CV curve was significantly increased (Figures 3B, F). The integral of the closed curve was positively correlated with the charge storage capacity. The larger the integral was, the better the capacitive performance of the electrode would be. We calculated the CSC of the electrode and found that the CSC of both electrode was significant increased (From 3.2 pC to 44.2 pC for Ni-Cr electrode and from 18.7 pC to 88.8 pC for CNT electrode) (Figures 3D, H), these phenomena probably due to the fact that the PEDOT film provide an effective activation zone for the interaction between the conductive polymer and the surrounding electrolyte. These results indicate that the electrochemical interface properties of PEDOT/SNP-MT coating electrodes are improved compared with bare electrodes.

### 3.3 Electrically controlled MT release

**FIGURE 4:**
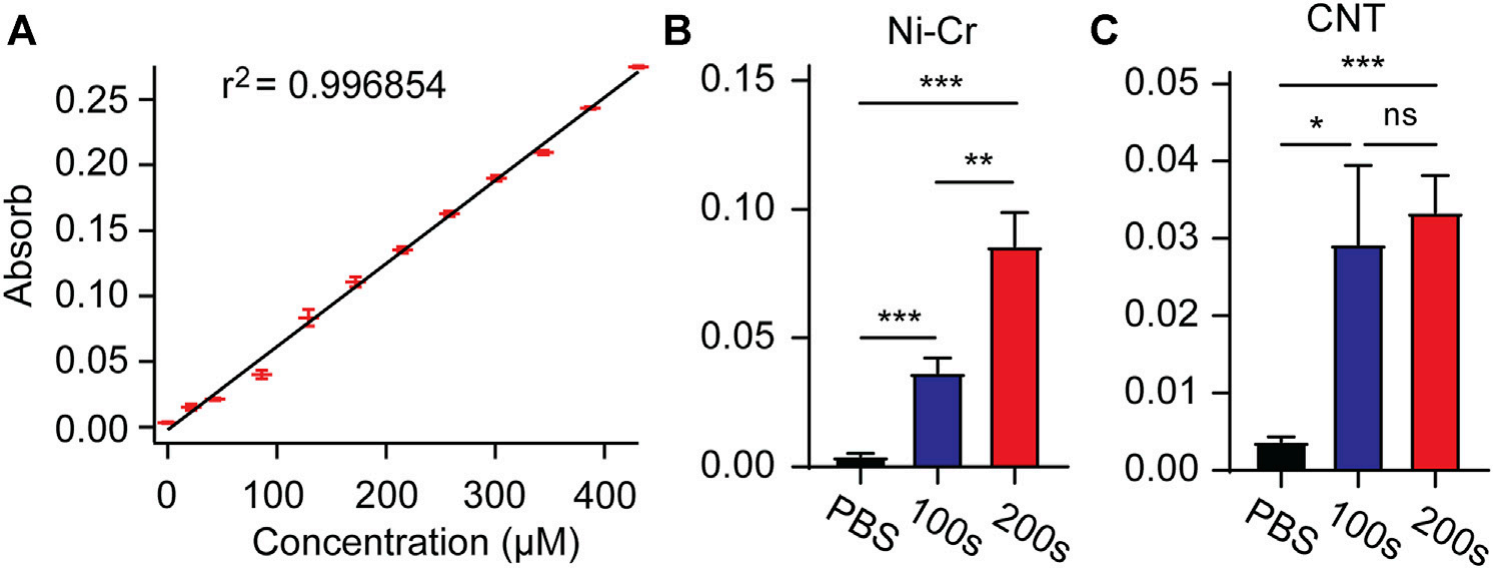
Electrically controlled MT release **(A)** Absorbance concentration absorption curve of standard solution. **(B)** The absorbance values after 100s and 200s stimulation of modified Ni-Cr alloy electrode. **(C)** The absorbance values after 100s and 200s stimulation of modified CNT electrode. These results confirmed that MT can be electrically controlled release by a unidirectional rectangular waveform stimulation. n = 9 for Ni-Gr alloy electrode, and n = 7 for CNT fiber electrode. Error bars show SEM. *p < 0.05, **p < 0.01, ***p < 0.005, Student’s T-Test.

Drug loading and release of electrically controlled conductive polymers provides an accurate method to deliver drugs directly to the implantation site as needed (Woeppel et al., 2019; Tan et al., 2021). After loading with MT in the electrode membrane, MT is released by a unidirectional rectangular waveform (5 ms, −10 µA, 100 Hz) into 250 μL of PBS. The concentration of the MT solution is measured by UV-visible absorption. After measurement of absorbance concentration absorption curve of standard solution, the solution concentration can be estimated according to the measured absorbance value (Figure 4A). We measured the absorbance values of modified Ni-Cr alloy electrode and CNT electrode after 100 s and 200 s stimulation, respectively. The results showed that compared with PBS (passive diffusion for 2 h), the stimulated drug release significantly increased the absorbance values, indicating that MT can be released from electrode (Figures 4B,C). Compared to 100 s stimulation, 200 s stimulation of modified Ni-Cr alloy electrode significantly increased the absorbance value, these results further confirmed that MT could be electrically controlled release by a unidirectional rectangular waveform stimulation. The observation of the electrode surface morphology after stimulation showed that the tips of the electrode still maintained the nanostructure.

### 3.4 Acute neural signal recording

**FIGURE 5:**
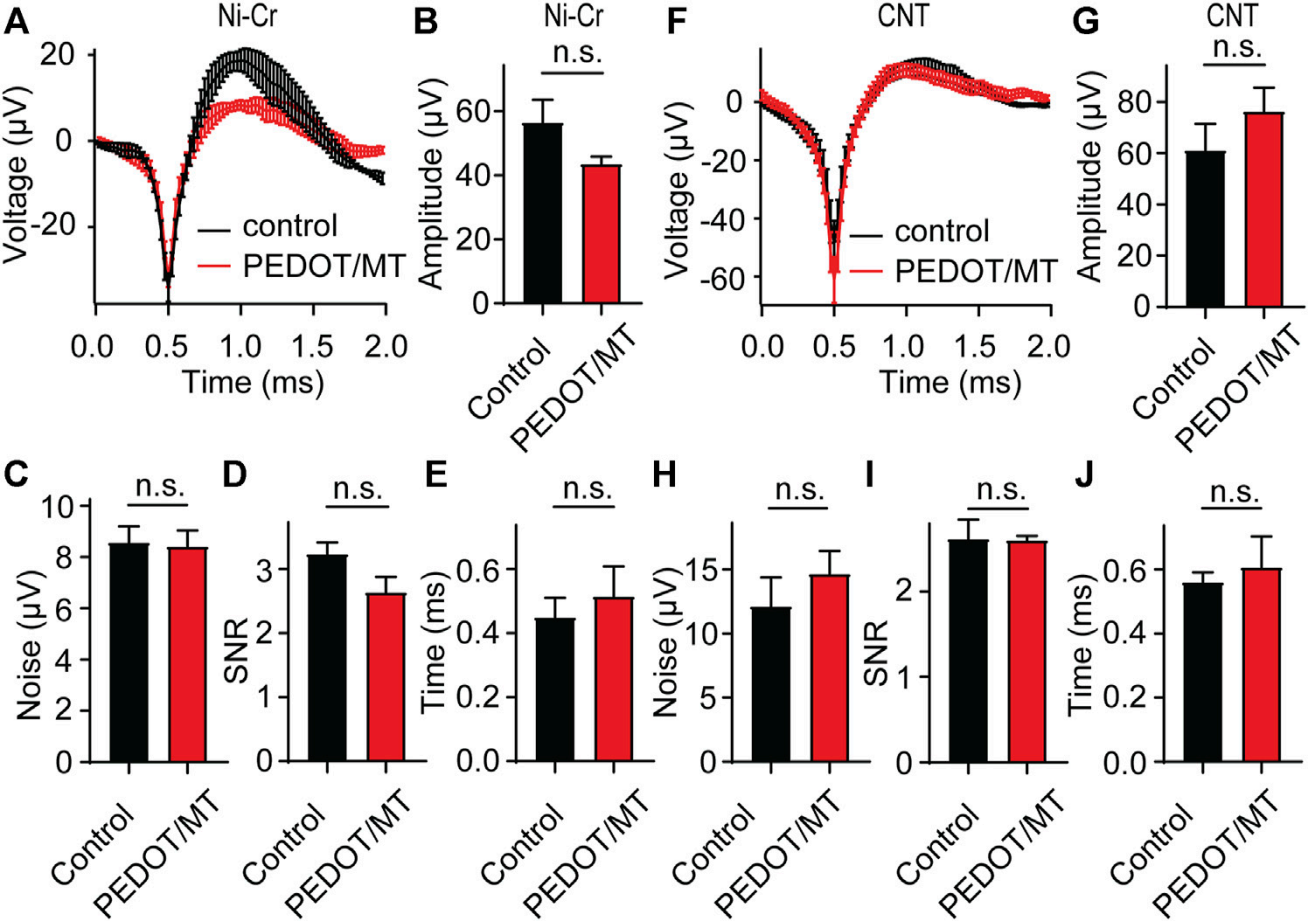
Acute neural signal recording **(A)** The acute nerve signal recording of the PEDOT/SNP-MT modified Ni-Cr alloy electrode (n = 5) and the electrode without any treatment (n = 6). **(B–E)** The spike amplitude, average noise level, signal to noise ratio (SNR) and valley-to-peak time of neural signals recorded by modified and unmodified Ni-Cr alloy electrode. **(F)** The acute nerve signal recording of the PEDOT/SNP-MT modified Ni-Cr alloy electrode (n = 7) and the electrode without any treatment (n = 7). **(G–J)** The spike amplitude, average noise level, signal to noise ratio (SNR) and valley-to-peak time of neural signals recorded by modified and unmodified Ni-Cr alloy electrode. These results indicate that the modified electrodes did not affect the collection of neural signals. Error bars show SEM, n.s., no significance, Student’s T-Test.

In order to explore the influence of modified materials on nerve signal acquisition, we implanted the PEDOT/SNP-MT modified electrodes and the untreated electrodes into the hippocampal CA1 area of rats for acute nerve signal recording and analysis. We compared the spike amplitude, average noise level, signal to noise ratio (SNR) and valley-to-peak time of neural signals recorded by modified and unmodified channels. As shown in Figure 5, after modification, there is no difference in the spike amplitude, average noise level, SNR and valley-to-peak time from both Ni-Cr alloy electrode and CNT electrode. These results indicate that the modified electrodes did not affect the collection of neural signals.

### 3.5 Chronic neural signal recording

**FIGURE 6:**
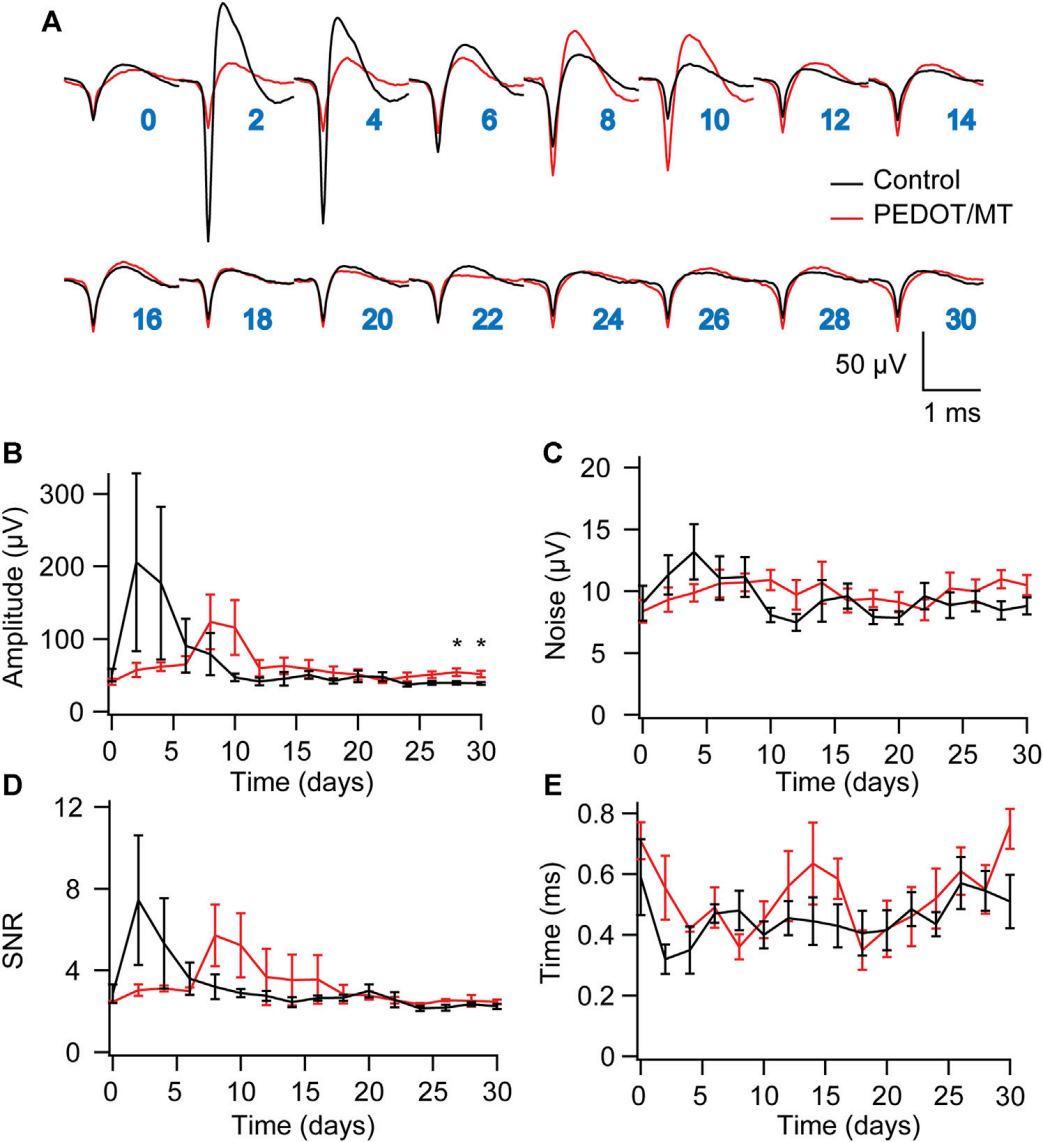
Chronic neural signal recording **(A)** Mean waveforms of units detected and isolated from day 1 to day 30 postimplantation with an electrode made of Ni-Cr alloy electrode. The waveforms are isolated and averaged from 2 to 8 min recording segments. **(B–E)** Valley-to-peak amplitude **(B)**, noise level **(C)**, SNR **(D)** and Valley-to-peak time **(E)** of the clustered single-units as a function of time. These results indicate that a more stable neural interface is formed between the modified electrode and brain tissue. n = 5. Error bars show SEM. *p < 0.05, Student’s T-Test.

**FIGURE 7:**
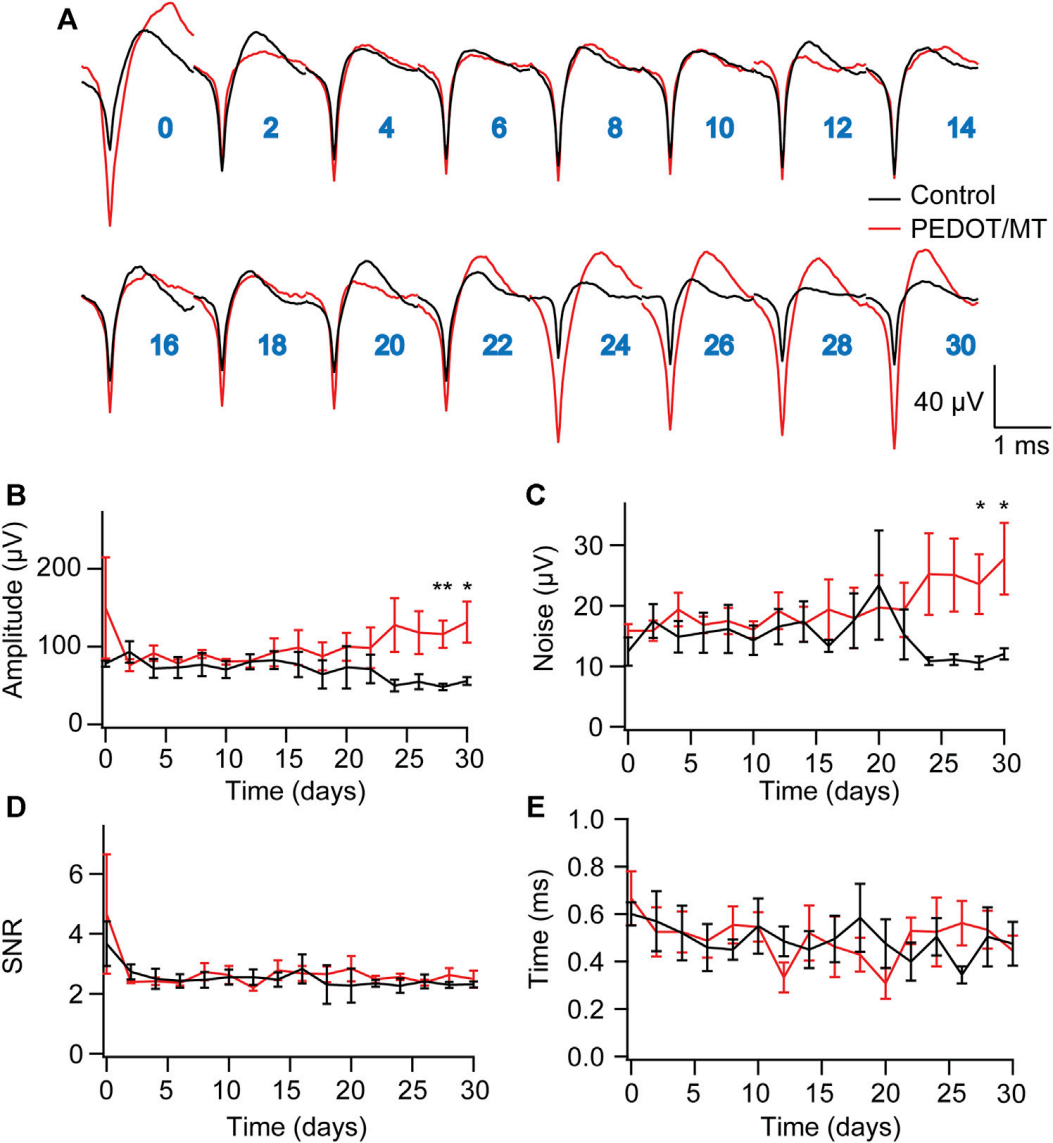
Chronic neural signal recording **(A)** Mean waveforms of units detected and isolated from day 1 to day 30 postimplantation with an electrode made of CNT fiber electrode. The waveforms are isolated and averaged from 2 to 8 min recording segments. **(B–E)** Valley-to-peak amplitude **(B)**, noise level **(C)**, SNR **(D)** and Valley-to-peak time **(E)** of the clustered single-units as a function of time. These results indicate that a more stable neural interface is formed between the modified electrode and brain tissue. n = 6 for modified electrode and n = 5 for control electrode. Error bars show SEM. *p < 0.05, **p < 0.01, Student’s T-Test.

Chronic reliable performance of implantable electrodes is critical for both neuroscience research and medical applications. However, after microelectrode implantation, the recording quality will be degraded over time due to glial encapsulation and neuron loss (Biran et al., 2005; Kozai et al., 2014; Kozai and Du, 2015). Typically, the deterioration in the recording quality happens over weeks and months (Nicolelis et al., 2003; Kozai and Jaquins-Gerstl, 2015). To test the chronic performance of the modified electrode, we implanted PEDOT/SNP-MT modified and unmodified electrodes into the hippocampal CA1 region of rats respectively. We examined the effect of electrically triggered MT release every other day on the quality and longevity of neural recording from implanted microelectrode in rat hippocampus for 1 month. All chronic recordings were performed on anesthetized animals and from spontaneous neuron firing. Supplementary Figures S3, S4 showed some representative chronic recording results with modified or unmodified Ni-Cr alloy electrode (Supplementary Figure S3) and CNT electrode (Supplementary Figure S4) implanted in the hippocampal. We found that Ni-Cr alloy electrode can stably and continuously detect single unit neural signals from rats within 1 month (Figure 6A). In addition, single-unit amplitude showed significant difference between the electrically controlled MT treated group and control animals after 28-day post-implantation (Figure 6B). However, there is no difference in average noise level, SNR and valley-to-peak time (Figures 6C–E). Furthermore, we also found that CNT electrode can stably and continuously detect single unit neural signals from rats within 1 month (Figure 7A). Single-unit amplitude showed significant difference between the electrically controlled MT treated group and control animals after 28-day post-implantation (Figure 7B). However, there is no difference in SNR and valley-to-peak time except that average noise level of CNT electrode is altered (Figures 7C–E). Interestingly, the spike amplitude of the electrically controlled MT treated group in CNT electrode showed gradually increase from 2-week post-implantation compared to the control (Figure 7B). This may be due to the cumulative effect of electrically triggered MT release every other day. These results indicate that a more stable neural interface is formed between the modified electrode and brain tissue.

### 3.6 Histological study of the response of brain tissue to chronic implantation of CNT fiber microelectrodes

**FIGURE 8:**
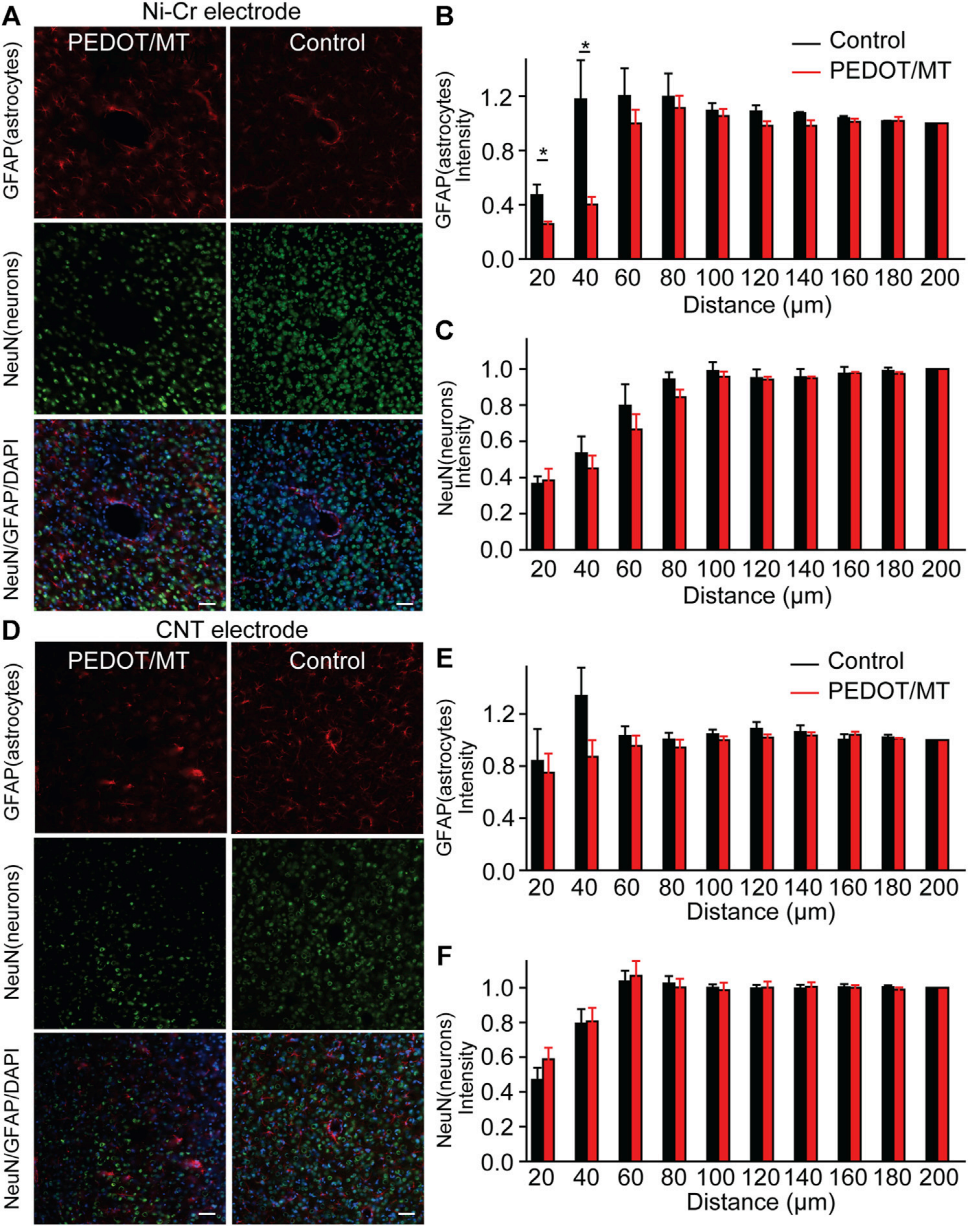
Histological studies of brain tissue reaction to chronically implanted Ni-Cr alloy microelectrodes and CNT microelectrodes **(A)** Immunofluorescence images of tissue responses following a 4 weeks implantation of an electrode made from Ni-Cr alloy electrode. The tissue was labeled for astrocytes (red), neurons (green), and nuclei (blue). Scale bar, 100 μm. **(B and C)** Normalized GFAP **(B)** and NeuN **(C)** fluorescence intensity as a function of distance from the center of the electrode tract, which is set as x = 0 μm. **(D)** Immunofluorescence images of tissue responses following a 4 weeks implantation of an electrode made from CNT electrode. The tissue was labeled for astrocytes (red), neurons (green), and nuclei (blue). Scale bar, 100 μm. **(E and F)** Normalized GFAP **(E)** and NeuN **(F)** fluorescence intensity as a function of distance from the center of the electrode tract, which is set as x = 0 μm. These histological data show that the modified electrode has the ability to improve and stabilize the microenvironment in the neural interface. It plays a role in the formation of glial scar around brain implants. n = 5. Error bars show SEM. *p < 0.05, Student’s T-Test.

The glial encapsulation and neuronal death near the recording electrode are considered to be the main factors that have a negative impact on the stability and longevity of neural signals collected by the electrode (McCreery et al., 2016; Golabchi et al., 2018; Lu et al., 2019). Tissue inflammation leads to neuronal death and the formation of glial scars, resulting in changes in the properties of the electrodes. To investigate the neuron and astrocyte distribution around the implanted area, NeuN combined with GFAP were stained 1 month after implantation to recognize neurons and astrocyte respectively (Figure 8). Quantitatively, the chronic electrically triggered MT release group yields almost 60.11% reduction for Ni-Cr alloy electrode and 25.69% reduction for CNT electrode in GFAP staining within 40 µm of electrode-tissue interface compared to the control group (Figures 8B and E), suggesting the chronic electrically triggered MT release is necessary to control the persistent inflammation. However, the number of neurons around the electrodes in the control and the chronic electrically triggered MT release group was almost the same (*p* < 0.05), suggesting that neither neuron recovery nor further neuron loss occurred in the chronic electrically triggered MT release group compared to the control group (Figures 8C, F). These histological data show that compared with the unmodified electrode, the modified electrode has the ability to improve and stabilize the microenvironment in the neural interface. It plays a role in the formation of glial scar around brain implants. These histological studies, together with neural signal recorded results, strongly indicate that a more stable brain computer interface is formed between PEDOT/SNP-MT modified electrodes.

## 4 Discussion

The implantation of brain-computer interface devices in the nervous system for the treatment of neurological diseases is becoming very common and long-term, high-quality nerve recording electrodes are becoming the focus of research (Zhang and Lieber, 2016; Ferro and Melosh, 2018; Pei and Tian, 2020; Kwon et al., 2021). Long-term stability of signal acquisition electrodes must meet some characteristics, such as high signal acquisition resolution, high electrochemical stability and good biocompatibility. In order to achieve these goals, many material innovations have been made in materials recently, usually focusing on shape factors (shape, thickness, weight, etc.) and mechanical properties. However, after the microelectrodes were implanted, the performance would decrease over time due to glial encapsulation and neuronal loss (Wang et al., 2018; Hejazi et al., 2021; Yang et al., 2021).

There are many treatments for inflammation and neuronal death. We chose MT because recent work has demonstrated that MT is capable of preserving neuronal health around implanted neural electrodes and maintaining high recording quality over time when systemically administered daily (Golabchi et al., 2018). Additionally, conducting polymers have been widely used for improving neural electrode recording performances by increasing effective surface area and reducing impedance (Ludwig et al., 2011; Woeppel et al., 2019; Yang et al., 2021). Therefore, we studied the novel PEDOT/SNP-MT modified electrode and examined the effect of electrically triggered MT release on the chronic performance in rat hippocampus. The electrical performance tests of nerve electrodes included electrochemical impedance and volt-ampere cycle curve. After PEDOT/SNP-MT coating, the electrochemical impedance at 1 kHz of the Ni-Cr alloy electrodes was significant reduced, while that of the CNT electrodes was significant increased (Figures 3A,C,E,G). The differences in impedance were likely resulted by the lower electronic conductivity of PEDOT/SNP-MT film than the CNT fibers. Despite the higher resistance, the CSC of both electrode was significant increased, which indicated that the characteristics of the electrodes produced by different surface modification methods were significantly different (Figures 3B,D,F,H). This may suggest that the electrochemical interface properties of PEDOT/SNP-MT coating electrodes are improved compared with bare electrodes as previously discussed (Woeppel et al., 2019; Tan et al., 2021).

In order to explore the influence of PEDOT/SNP-MT film on nerve signal acquisition, we recorded and analyzed the acute and chronic nerve signal (Figures 5–7). Animals were anesthetized during the recording process to eliminate motion artifacts. We compared the spontaneous individual unit activity recorded by electrodes implanted in rat hippocampus and found that the modified electrodes did not affect the collection of neural signals (Figure 5). Interestingly, single-unit amplitude during chronic recording showed significant difference between the electrically controlled MT treated group and control animals after 28-day post-implantation (Figures 6, 7). The results presented here suggested that chronic MT treatment increases the quality (spike amplitude) of the recording behavior of implanted electrodes. As a drug delivery platform, the drug can be controllably released from PEDOT/SNP which can protect the activity of the drug (Woeppel et al., 2019; Tan et al., 2021). In terms of neural interface applications, our data for the control group showed similar trends in recording behavior that have been reported before, and our research confirmed that the released MT can maintain chronic recording quality (Golabchi et al., 2018). Over the initial 2 weeks after implantation, the recording amplitude continued to drop, which corresponds to the most dynamic changes in tissue during the injury and wound healing phase. Interestingly, the spike amplitude of the electrically controlled MT treated group in CNT electrode showed gradually increase from 2-week post-implantation. Compared with the results of intraperitoneal injection that the spike amplitude remained stable in the following weeks, the difference may due to the effective amount of MT in the recording site (Golabchi et al., 2018).

Previous studies have shown the persistent presence of microglia/astrocyte near the implants (Golabchi et al., 2018; Lu et al., 2019). In addition, it is reported that continuous leakage of blood-brain barrier will further aggravate inflammation and lead to neuron loss and demyelination (Hejazi et al., 2021; Yang et al., 2021). Recent reports have proved the effective role of MT therapy in maintaining high-quality electrode-tissue interface, and indicated that MT may promote neuroprotection through its anti-apoptosis, anti-inflammatory and antioxidant properties (Golabchi et al., 2018). Histological analysis provides further insight into these biological mechanisms. GFAP-labeled reactive astrocytes are a good indicator of reactive glial hyperplasia in the nervous system damage. Our results showed that MT treatment reduced the expression of GFAP in the electrode tip (Figure 8). These data suggested that the chronic electrically triggered MT release inhibited the activation of astrocytes. In addition, we performed additional staining and imaging to check for shallower depths. Intensity analyses for GFAP are shown in Supplementary Figure S5. MT treatment did not show any significant differences in GFAP expression in the apical region of the brain compared to controls. These results suggest that MT release was restrict in the local electrode tip region. By combining the electrophysiological recorded data with histological analysis, this provides a potent biological factor that play a critical role in the chronic recording quality. This may suggest that the electrochemical interface properties of PEDOT/SNP-MT coating electrodes are improved compared with bare electrodes and the electrode-tissue interface became stabilized from the physical barrier perspective as previously discussed (Woeppel et al., 2019; Tan et al., 2021).

The results of this study provide evidence of PEDOT/SNP-MT coating electrode in improving the chronic intracranial recording performance of the electrode. Conducting polymers are at the forefront of biomaterials research with applications in brain-computer interface and drug delivery systems. In summary, by combining the electrophysiological recorded data with histological analysis, we examined the effect of electrically triggered MT release every other day on the quality and longevity of neural recording from implanted microelectrode in rat hippocampus for 1 month. These results demonstrate the potent effect of PEDOT/SNP-MT treatment in improving the quality of chronic neural recording possibly through its anti-inflammatory property. Additional histological staining, in particular for staining for microglia, ROS damage, caspase-3 are necessary to further strengthen the argument that MT release from the electrode site had a beneficial effect on the surround tissues. It can be concluded that continuous electrically triggered local MT release may be a potential system to improve the quality of long-term recording, meanwhile, it is necessary to develop method with abundant drug loading technology.

## Data Availability

The original contributions presented in the study are included in the article/Supplementary Material, further inquiries can be directed to the corresponding authors.
