# Supplementary material for: On-demand electrically controlled melatonin release from PEDOT/SNP composite improves quality of chronic neural recording
Source: Front Bioeng Biotechnol. 2023 Nov 15;11:1284927. doi: 10.3389/fbioe.2023.1284927 (PMC10684936; doi:10.3389/fbioe.2023.1284927)
Supplement: Supplementary file 1 [file Table1.DOCX]

Supplementary Material

# Supplementary Figures


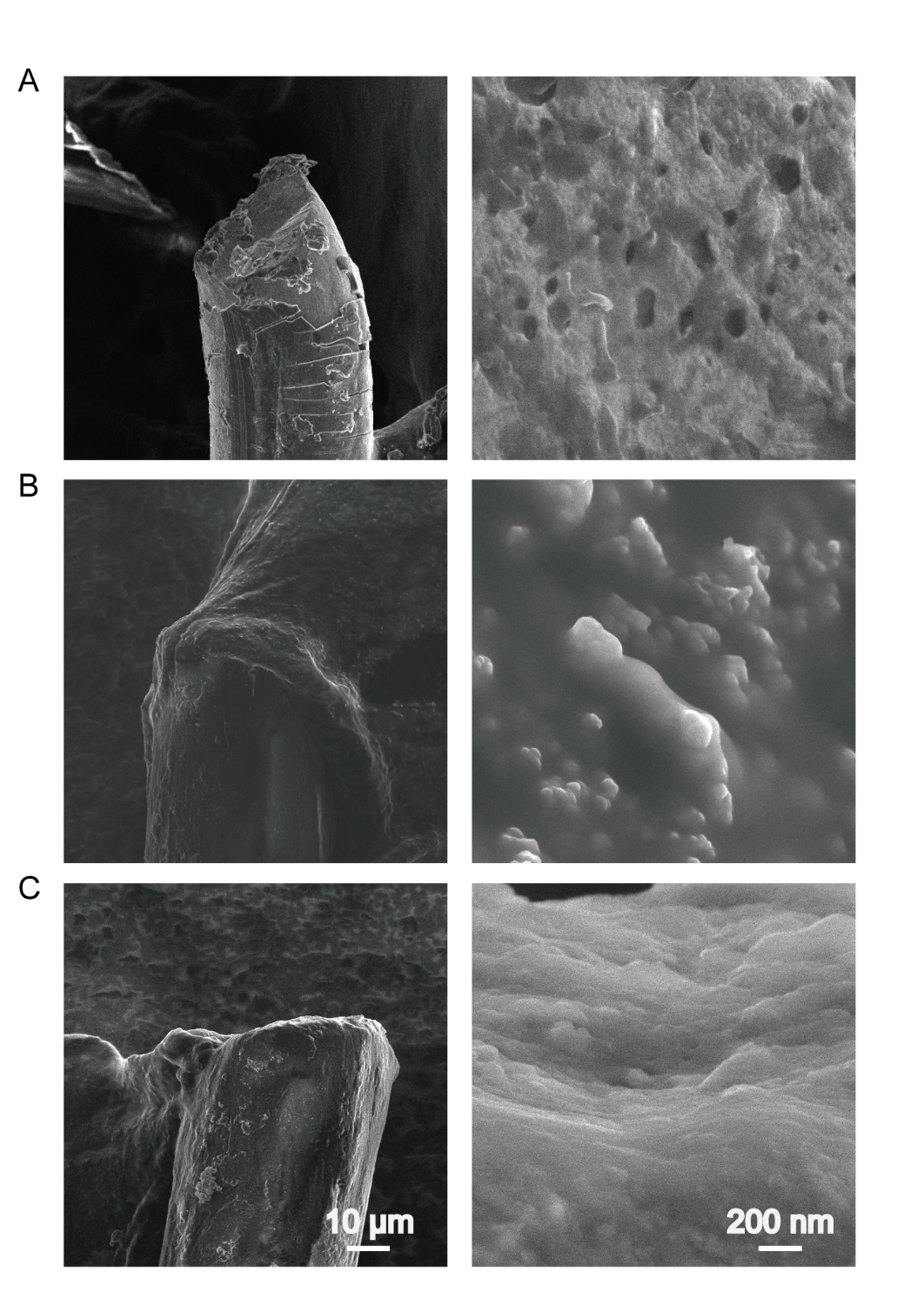


**Supplementary Figure 1.** Characterization of sharpened Ni-Cr alloy electrodes coated with PEDOT/SNP-MT (A) Bare Ni-Cr alloy electrode without any treatment. (B) Ni-Cr alloy electrode coated with PEDOT/SNP-MT before electrically stimulation. (C) Ni-Cr alloy electrode coated with PEDOT/SNP-MT after 200s electrically stimulation.


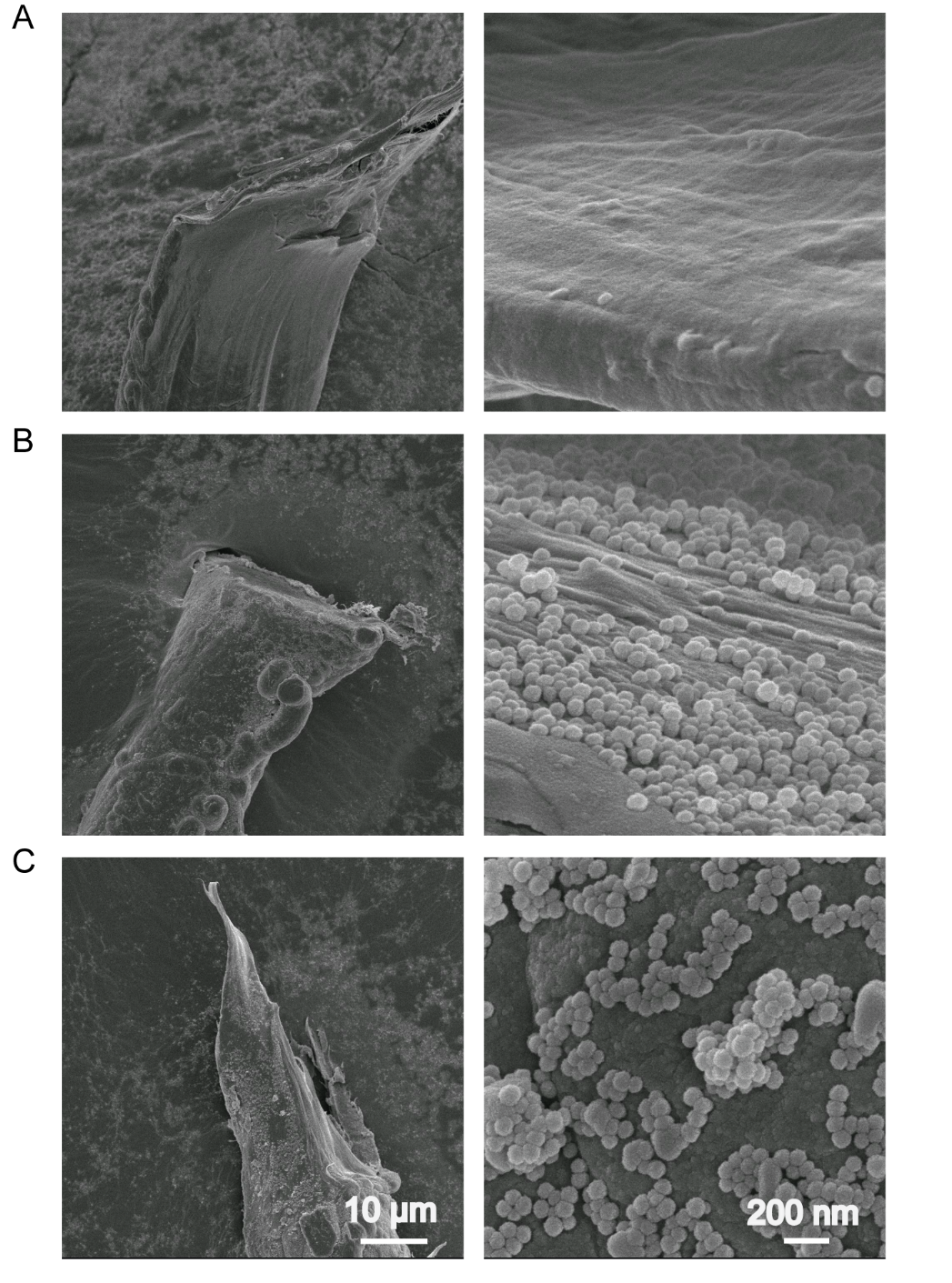


**Supplementary Figure 2.** Characterization of sharpened CNT electrodes coated with PEDOT/SNP-MT (A) Bare CNT electrode without any treatment. (B) CNT electrode coated with PEDOT/SNP-MT before electrically stimulation. (C) CNT electrode coated with PEDOT/SNP-MT after 200s electrically stimulation.


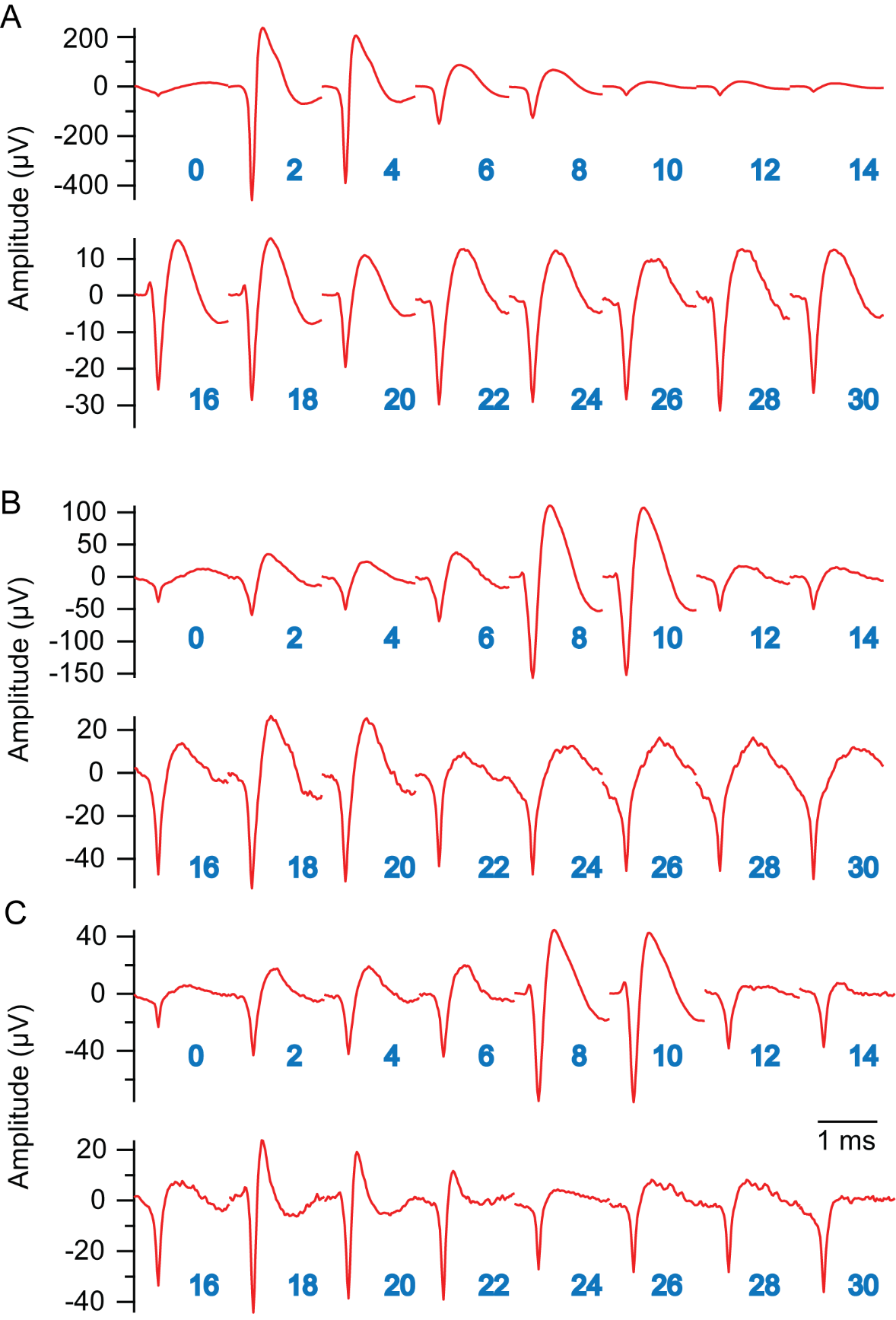


**Supplementary Figure 3.** Three examples of chronic neural recording with Ni-Cr alloy electrodes (A) One example of neural recording from control rat. Note that large signals were generated on the second and fourth days. (B and C) Two examples of neural recording with PEDOT/SNP-MT coated electrodes. Note that large signals were generated on the eighth and tenth days.


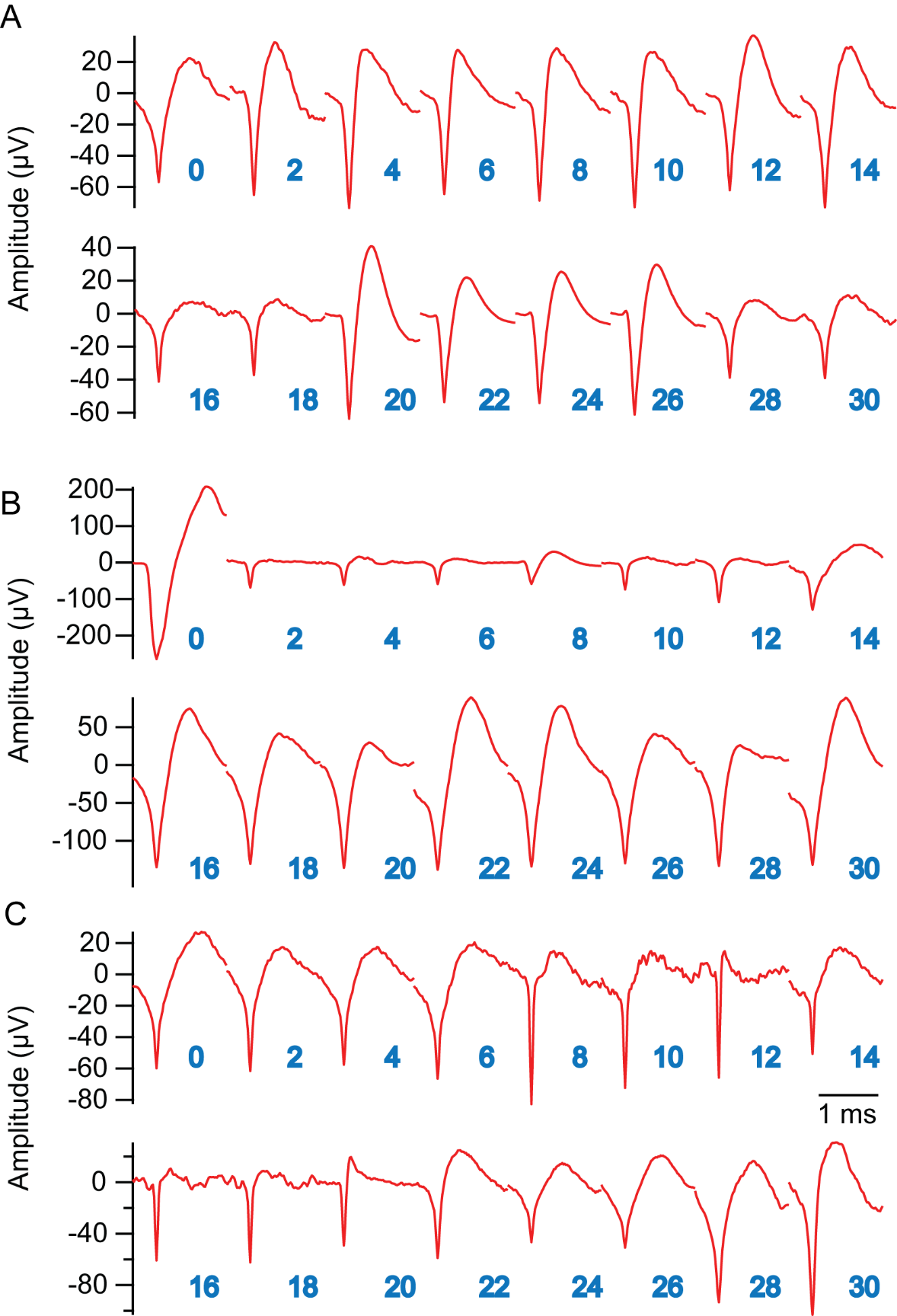


**Supplementary Figure 4.** Three examples of chronic neural recording with CNT electrodes (A) One example of neural recording from control rat. (B and C) Two examples of neural recording with PEDOT/SNP-MT coated electrodes.


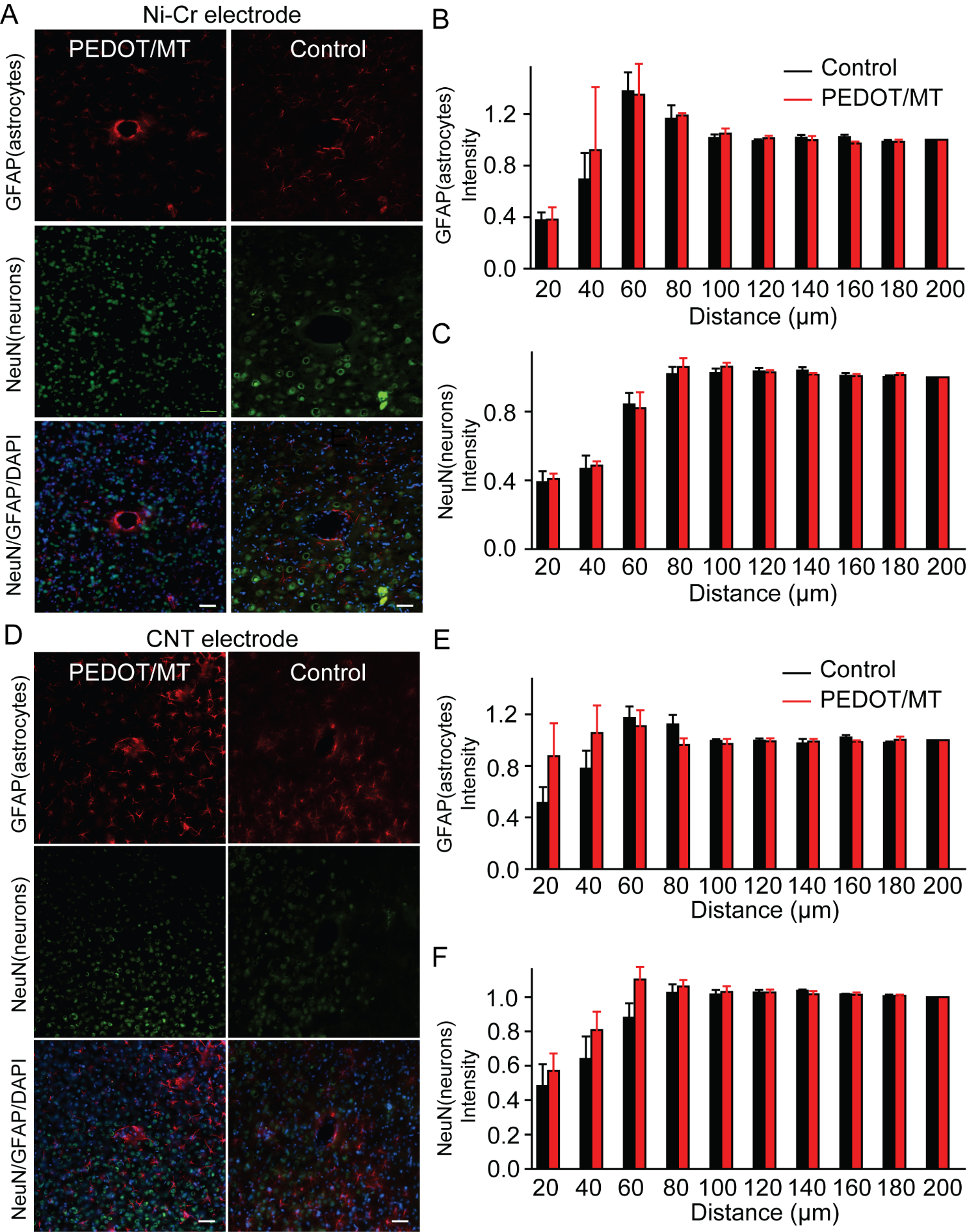


**Supplementary Figure 5.** Additional staining and imaging to examine shallower depths implanted by Ni-Cr alloy electrode and CNT electrode (A) Immunoﬂuorescence images of tissue responses following a 4 week implantation of an electrode made from Ni-Cr alloy electrode. The tissue was labeled for astrocytes (red), neurons (green), and nuclei (blue). Scale bar, 100 μm. (B and C) Normalized GFAP (B) and NeuN (C) ﬂuorescence intensity as a function of distance from the center of the electrode tract, which is set as x =0 μm. (D) Immunoﬂuorescence images of tissue responses following a 4 week implantation of an electrode made from CNT electrode. The tissue was labeled for astrocytes (red), neurons (green), and nuclei (blue). Scale bar, 100 μm. (E and F) Normalized GFAP (E) and NeuN (F) ﬂuorescence intensity as a function of distance from the center of the electrode tract, which is set as x =0 μm. Error bars show SEM.


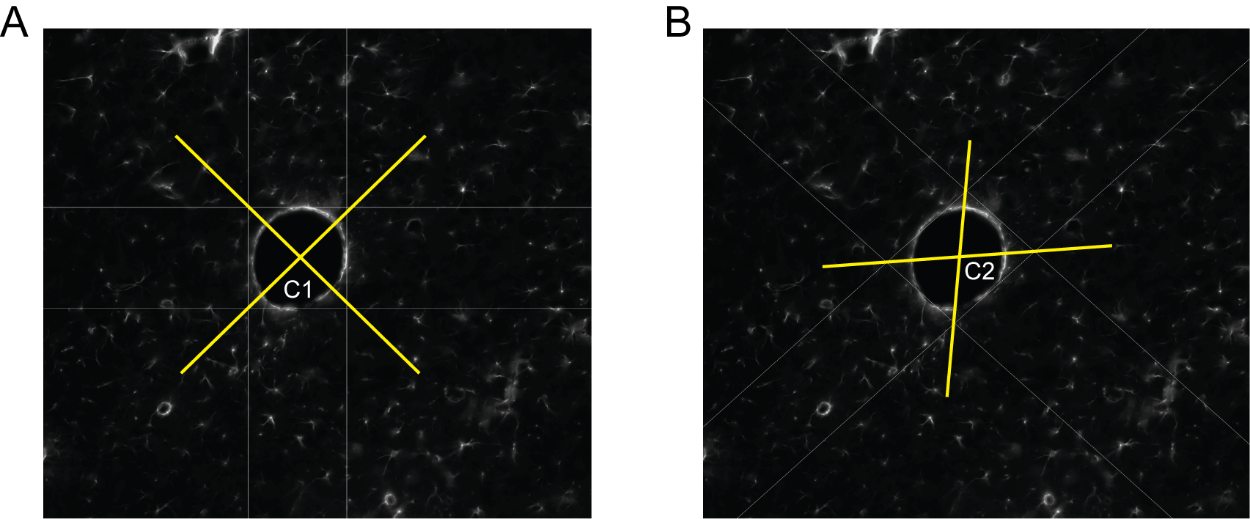


**Supplementary Figure 6.** Procedure of defining the center of the probe tract (x=0 μm) for histology studies (A) Two horizontal lines and two vertical lines that are tangent to the hole outline were drawn, which forms a rectangle. The intersection of the diagonals is set as point C1. (B) Four 45° angle lines that are tangent to the hole outline were drawn, which forms another rectangle. The intersection of the diagonals is set as point C2. The midpoint of C1 and C2 is defined as the center of the probe tract (x=0 μm).
